# Methane and Nitrous Oxide Flux after Biochar Application in Subtropical Acidic Paddy Soils under Tobacco-Rice Rotation

**DOI:** 10.1038/s41598-019-53044-1

**Published:** 2019-11-21

**Authors:** Yibin Huang, Chengji Wang, Cheng Lin, Yushu Zhang, Xi Chen, Lina Tang, Cenwei Liu, Qingrong Chen, Mabel Ifeoma Onwuka, Tieying Song

**Affiliations:** 1 Institute of Soil and Fertilizer, Fujian Academy of Agricultural Sciences, Fuzhou, 350013 P.R. China; 2 Agricultural Ecology Institute, Fujian Academy of Agricultural Sciences, Fuzhou, 350013 P.R. China; 3 Institute of Biotechnology, Fujian Academy of Agricultural Sciences, Fuzhou, P.R. China; 4 Tobacco Agriculture Science Research Institute of Fujian Province, Fuzhou, 350003 P.R. China; 5 College of Crop and Soil Sciences, Michael Okpara University of Agriculture Umudike, PMB 7267 Umuahia, Abia State Nigeria

**Keywords:** Carbon cycle, Element cycles

## Abstract

Biochar amendment is a good means of mitigating methane (CH_4_) and nitrous oxide (N_2_O) emissions. However, the effects of biochar amendment on N_2_O and CH_4_ reduction in soil under rotation with different soil moisture contents is not well understood. To understand CH_4_ and N_2_O flux from soil with biochar amendment under water-unsaturated and water-saturated conditions, a field experiment was conducted in a tobacco-rice rotation field in subtropical China to investigate N_2_O and CH_4_ emissions following soil amendment with tobacco straw biochar at rates of 0, 10, 40 and 80 t·ha^−1^ (B0, B10, B40 and B80, respectively). N_2_O and CH_4_ emissions were monitored by a closed-chamber method in the water-unsaturated tobacco (UT) and water-saturated rice (SR) seasons during the 2015 planting season. The soil pH increased from 5.4 in the control to 6.1 in the soil amended with biochar at 80 t·ha^−1^ in the UT season. During both the UT and SR seasons, with biochar amendment at 40 and 80 t·ha^−1^, the soil bulk density (BD) decreased, while the soil organic matter (SOM) and available potassium (Av. K) contents increased. N_2_O flux was significantly greater in UT than in SR in the controls but decreased with the application of biochar during both the UT and SR seasons. The cumulative CH_4_ emission decreased with the rate of biochar application and the methanotroph *pmoA* gene copy number in soils and increased with the methanogenic archaea 1*6Sr DNA* gene copy number in soils during the rice-cropping season. These results indicated that biochar amendment could decrease methanogenic archaea and increase of methanotroph *pmoA* gene, which are the mechanistic origin for CH_4_ reduction.

## Introduction

CH_4_ and N_2_O are important greenhouse gases in the atmosphere^1,2^. Due to human activities, the concentrations of N_2_O and CH_4_ in the atmosphere increased from 270 ppbv and 324 ppb in 1750 to 324 ppbv and 1803 ppb in 2011, respectivelly^2,3^. With the growing demand for food, further increases in these greenhouse gasses are projected in the future^3^. Agricultural soil is the main source of N_2_O and CH_4_ emissions, accounted for approximately 66% and 50% of total emissions, respectively^1,4^. To mitigate global warming, it is necessary to employ strategies that will reduce these gas emissions from agricultural soil^5^.

Reducing N fertilizer input and increasing its use efficiency could decrease N_2_O emissions; biochar application might play an important role in this approach. With its feedstock availability and favourable properties, biochar has been considered a good input for improving crop N use efficiency and increasing carbon (C) return to soil^6–8^. A meta-analysis using data across 208 peer-reviewed studies showed that symbiotic biological N_2_ fixation and plant N uptake were increased by 63% and 11%, respectively, with biochar amendment^9^. It has been found that the crop growth response with biochar is greater in acidic soils than neutral and alkaline soils because the soil nutrient-retention capacity is typically low in acidic soil and applying biochar will increase this capacity^10^. Thus, there is an increase in crop growth as the pH of soil amended with biochar is increased^11^. However, the rate of nitrification and denitrification is improved in acidic soil with increasing pH, and these are the main pathways for N_2_O production. Conversely, as the soil pH is increased, the activity of N_2_O reductase (N_2_O-R) is improved^12^. This improvement will lead to more reduction of N_2_O to N_2_ (which is the last step of the denitrification process), resulting in a decrease in the N_2_O emission rate. Additionally, biochar can reduce *nirK* abundance and increase *nosZ* abundance, which will inhibit N_2_O production and simultaneously increase N_2_O consumption in acidic soil^13,14^. Therefore, the effects of biochar on N_2_O emissions in acidic soil may be different from those in other soil types.

CH_4_ is primarily produced in an exclusively anaerobic process by methanogenic archaea^4,15^. However, under unsaturated conditions, soils are considered sinks for CH_4_^7^. Powlson *et al*.^16^ reported that non-flooded upland soils are an important CH_4_ sink and that approximately 15% of global CH_4_ oxidation is attributed to this sink. Microbial oxidation of CH_4_ is the main pathway of soil CH_4_ uptake, which is driven by methanotrophs. Some methanotrophs feed solely on CH_4_, while others are facultative methanotrophs, which include *Methylocella* and *Methylocapsa*^17,18^. It has been reported that CH_4_ uptake in soil may be increased by biochar amendment due to the adsorption of CH_4_ onto the surfaces of biochar^19^. Additionally, soil aeration may be affected when biochar is added, and this may increase diffusive CH_4_ uptake^20^. Biochar has also been observed to stimulate methanotrophic CH_4_ consumption in anoxic environments, particularly at oxic/anoxic interfaces^21^. For example, most CH_4_ is produced in anoxic sediment in saturated soils, and some CH_4_ is consumed at the aerated root interface^8^. It was reported by Feng *et al*.^22^ that under saturated conditions, biochar significantly increased the strength of CH_4_ sink properties compared to the controls via decreasing the ratio of methanogenic archaea to methanotrophic bacteria^8^. Furthermore, biochar can provide a C substrate for microbial CH_4_ oxidation in soils^20^, and the labile C of biochar may be used as a methanogenic substrate in anoxic environments, promoting CH_4_ production^23^. However, the effects of biochar on CH_4_ emissions from soils under saturated and unsaturated conditions are not fully understood.

Additionally, uncertainty exists as to whether biochar’s GHG mitigation effects persist for one cropping season or longer. Lentz *et al*.^24^ suggested that the GHG mitigation effects of biochar application may be long-lived, whereas Spokas^25^ indicated that they were mainly short-term (a few days to several weeks). Therefore, further study is required to determine the period of GHG mitigation effects resulting from biochar application.

Tobacco followed by rice cropping is a common agricultural practice in South China. However, tobacco straw is discarded beside the field after harvest, and this may lead to disease outbreaks and infections. Instead of simply discarding tobacco straw, incorporating this material into the soil as biochar has been widely recommended. This will help improve soil organic matter and reduce environmental pollution caused by straw littering.

To investigate the effect of biochar made from tobacco straw on N_2_O and CH_4_ emissions in acidic soil under rotation with different water regimes, a field experiment with tobacco and rice rotations was conducted in subtropical China. Specifically, we hypothesize that soil amendment with biochar (i) may not reduce the N_2_O emission rate, given that stimulating N_2_O reduction may be counteracted by improved nitrification and denitrification in acidic soil due to increased soil pH after biochar application, and (ii) affects the emissions of N_2_O and CH_4_ early in the first planting season but not in the second planting season because the active surfaces of the biochar become saturated over time.

## Results

### Soil properties

The soil pH determined after tobacco harvest increased with an increased rate of biochar application; the highest pH was found in B80 (Table 1). However, no significant differences were recorded in pH after the rice harvest (Table 1). Soil BD was decreased from 1250.0 kg·m^−3^ in the control to 1170.0 kg·m^−3^ and 1160.0 kg·m^−3^ in B40 and B80 in the UT season, and from 1360.0 kg·m^−3^ in the control to 1210.0 kg·m^−3^ and 1190.0 kg·m^−3^ in B40 and B80 in the SR season. However, the SOM and Av. K contents increased with increasing rates of biochar in both the UT and SR seasons.

**Table 1:** Soil properties after biochar amendment.

Treat- ment	UT season						SR season					
	pH	SOM g·kg^−1^	BD kg·m^−3^	Av. N mg·kg^−1^	Av. P mg·kg^−1^	Av. K mg·kg^−1^	pH	SOM g·kg^−1^	BD kg·m^−3^	Av. N mg· kg^−1^	Av. P mg·kg^−1^	Av. K mg·kg^−1^
B0	5.4c	22.7b	1250.0 a	122.2a	34.9a	117.0b	5.5a	22.8b	1360.0ab	111.5b	24.6b	103.4c
B10	5.6bc	25.5b	1210.0ab	127.3a	37.3a	148.8b	5.4a	24.5b	1310.0 a	120.9b	46.6a	137.8c
B40	5.6bc	32.2a	1170.0bc	122.2a	36.3a	303.3b	5.2a	30.2a	1210.0 b	121.7b	51.0a	385.6b
B80	6.1a	35.3a	1160.0 c	120.3a	39.5a	481.3a	5.6a	34.9a	1190.0 b	153.0a	51.5a	467.6a

The soil samples were collected after tobacco and rice were harvested. Data in the table are the means, and different letters indicate significant differences at 5%; B0, B10, B40 and B80 are no biochar applied and biochar applied at the rate of 10, 40 and 80 t·ha^−1^, respectively; UT is unsaturated tobacco cropping; SR is saturated rice cropping; ccSOM is soil organic matter; BD is bulk density; Av. N is alkali-hydrolysable nitrogen; Av. P is available phosphorus; and Av. K is available potassium.

### N_2_O and CH_4_ emissions

**Figure 1:**
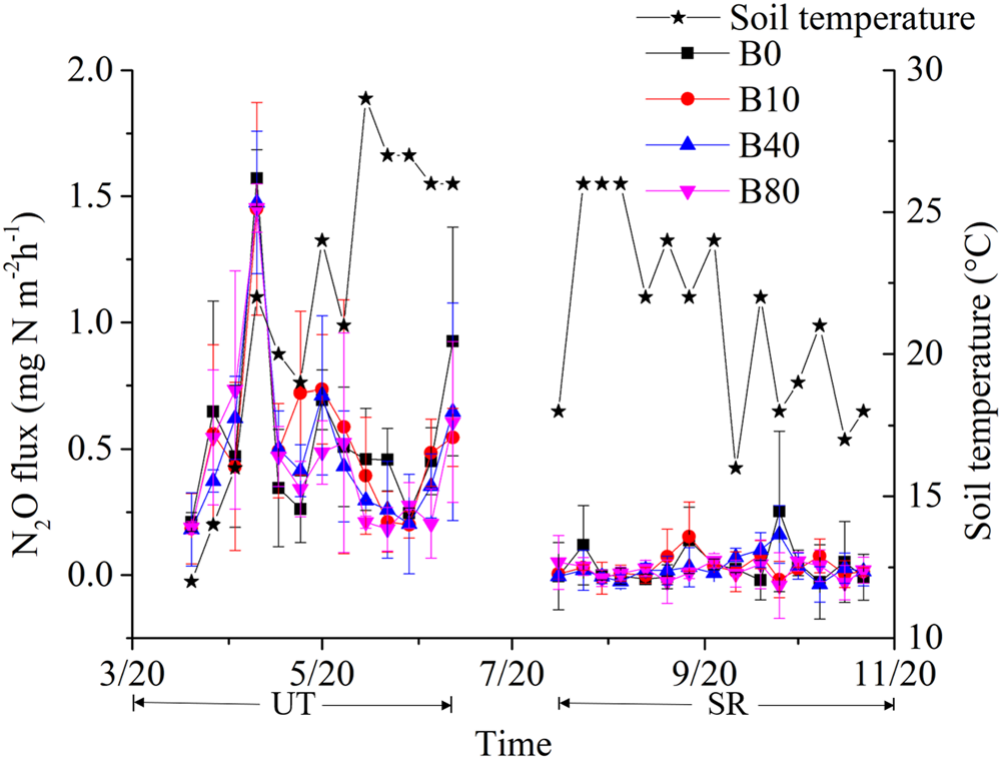
Temporal pattern of N_2_O fluxes for the different treatments during the tobacco and rice growth periods. The period from 3/20 (MM/DD) to 7/20 was the unsaturated tobacco growth season (UT), and the period from 7/20 to 11/20 was the saturated rice growth season (SR); B0, B10, B40 and B80 are no biochar applied and biochar applied at a rate of 10, 40 and 80 t·ha^−1^, respectively.

**Figure 2:**
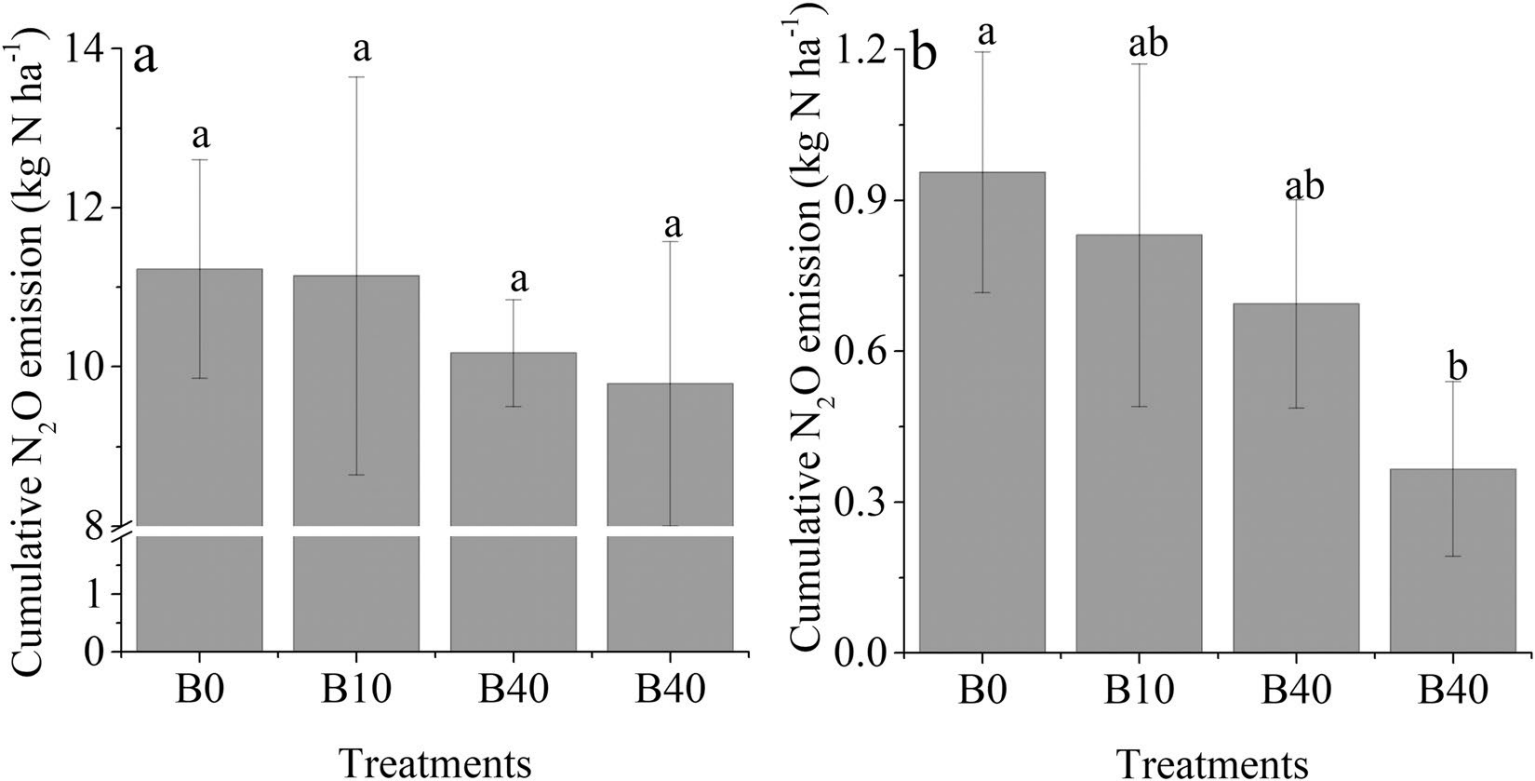
Cumulative N_2_O missions during the tobacco (**a**) and rice (**b**) growth periods. Values with same letter are not significantly different (p < 0.05); B0, B10, B40 and B80 are no biochar applied and biochar applied at the rate of 10, 40 and 80 t·ha^−1^, respectively.

**Figure 3:**
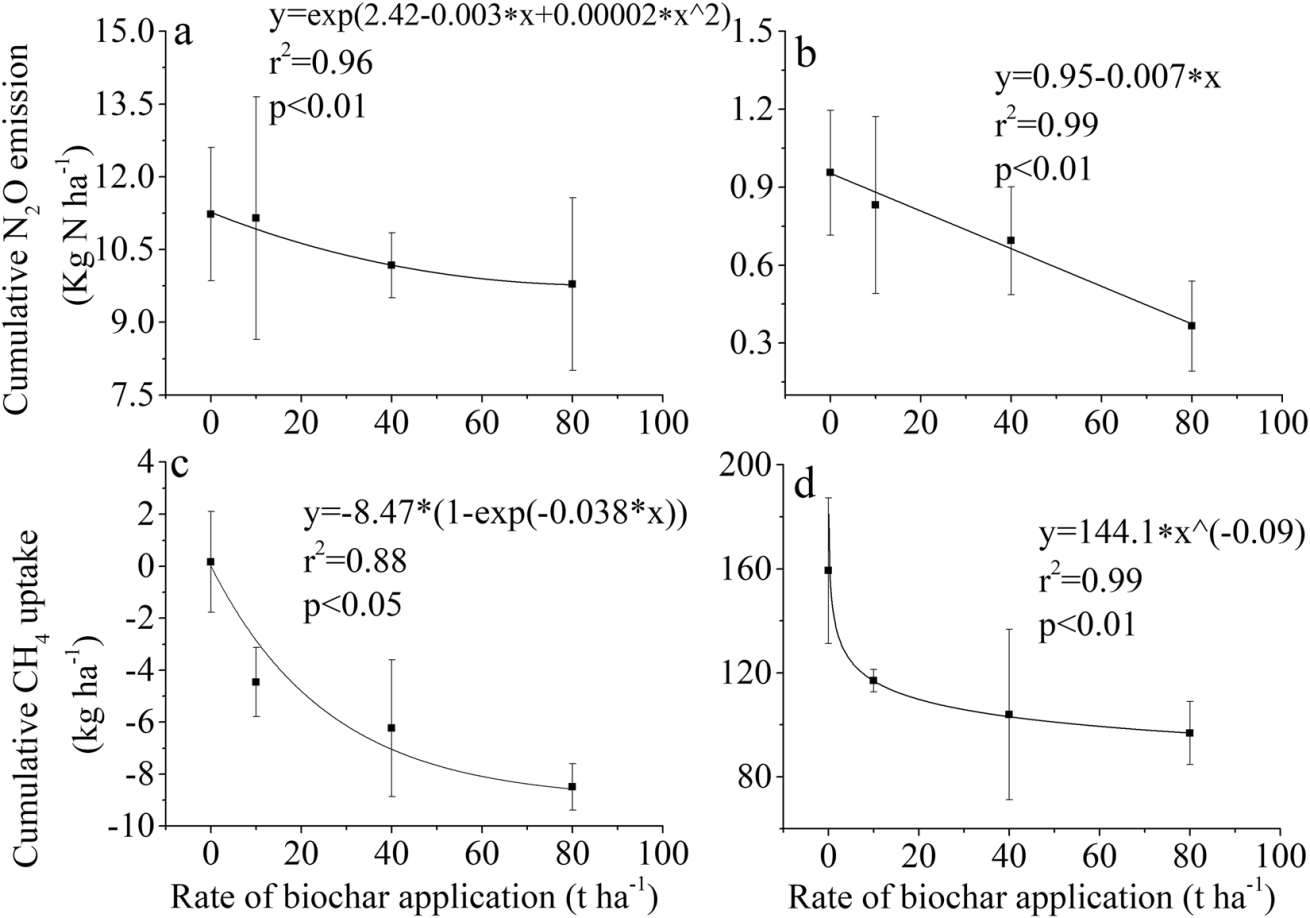
Relationship between the rate of biochar application and cumulative N_2_O emission during the tobacco (**a**) and rice (**b**) growth periods and between the rate of biochar application and cumulative CH_4_ uptake during the tobacco growth season (**c**) and emission during the rice growth period (**d**). Positive values of CH_4_ flux are CH_4_ emission, and negative values are CH_4_ uptake.

The N_2_O flux from all treatments was greater during the UT season than in the SR season (Fig. 1). The highest rate of N_2_O emission in most of the flux peaks was found in the B0 treatment during the experiment (Fig. 1). The cumulative N_2_O emissions during the UT season was 9.8 to 11.3 kg N·ha^−1^, which was significantly higher than that during the SR season by ≈10 times (Fig. 2). There was no significant difference among the treatments during the UT season (Fig. 2a), whereas the cumulative N_2_O flux during the SR season from B80 was significantly lower than that from B0 (Fig. 2b). There was a negative relationship between cumulative N_2_O flux and the rates of biochar applied during the UT and SR seasons (Fig. 3a,b, respectively).

**Figure 4:**
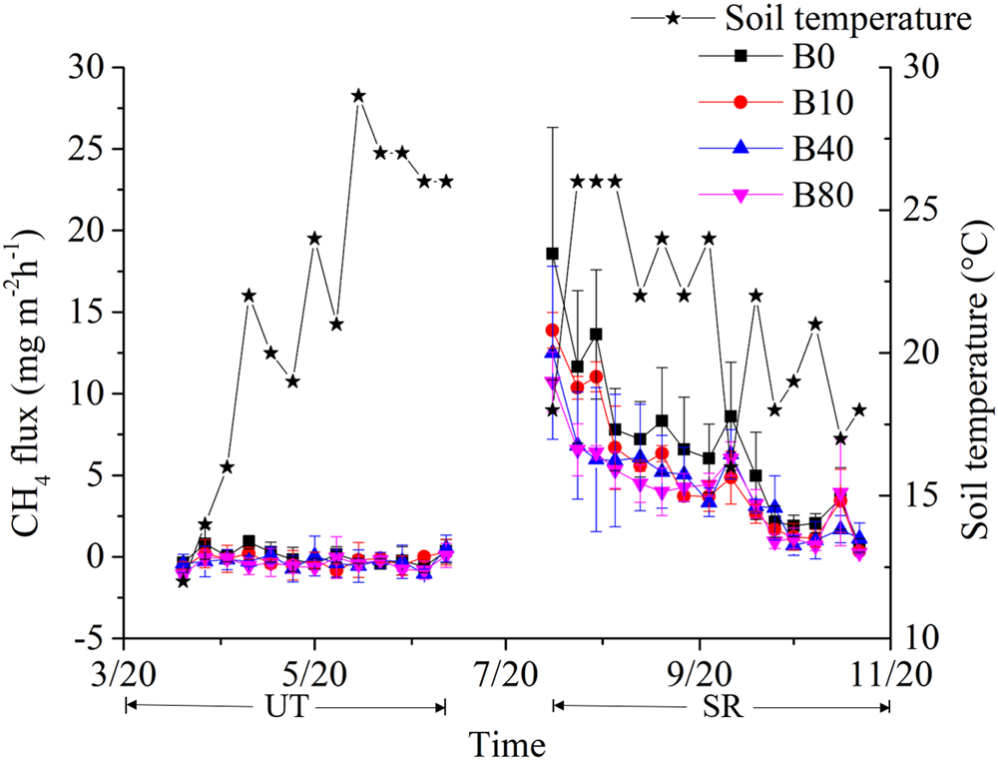
Temporal pattern of CH_4_ fluxes for the different treatments during the tobacco and rice growth periods. The period from 3/20 (MM/DD) to 7/20 was the unsaturated tobacco growth season (UT), and the period from 7/20 to 11/20 was the saturated rice growth season (SR); B0, B10, B40 and B80 are no biochar applied and biochar applied at the rate of 10, 40 and 80 t·ha^−1^, respectively.

**Figure 5:**
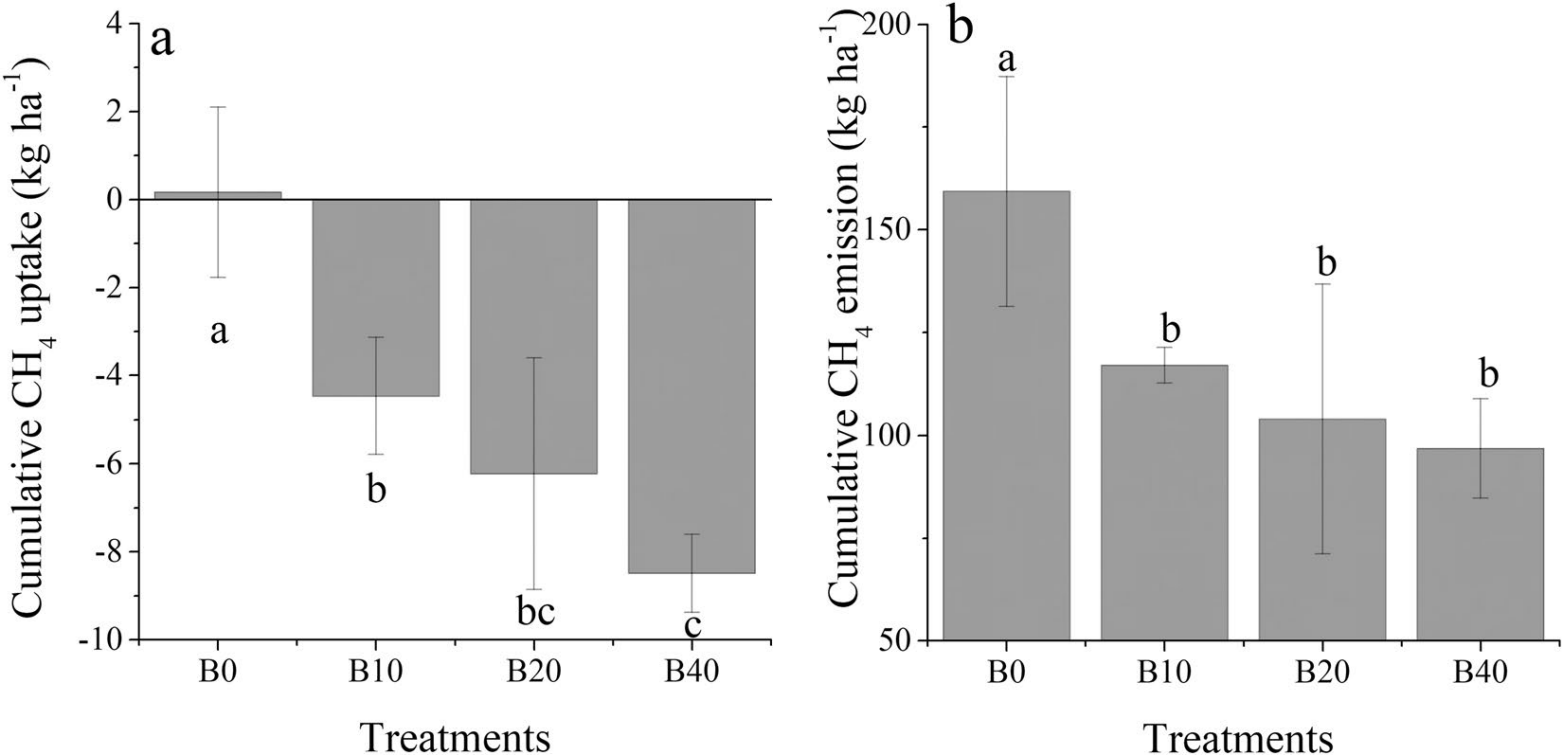
Cumulative CH_4_ uptake during the tobacco growth season (**a**) and CH_4_ emissions during the rice growth period (**b**). Values with the same letter are not significantly different (p < 0.05); B0, B10, B40 and B80 are no biochar applied and biochar applied at the rate of 10, 40 and 80 t·ha^−1^, respectively. Positive values of CH_4_ flux are CH_4_ emission, and negative values are CH_4_ uptake.

Unlike N_2_O, CH_4_ was taken up during the UT season, and the flux was also recorded during the SR season (Fig. 4). In the first two months of the SR season, the greatest CH_4_ emission flux was observed for B0, followed by B10, B40 and B80 (Fig. 4). This indicated that CH_4_ was affected by the rate of biochar amendment. The cumulative CH_4_ uptake during the UT season in the B80 treatment was −8.5 kg·ha^−1^, which was significantly greater than the values obtained for the B0 and B10 treatments (Fig. 5a). The cumulative CH_4_ emission during the SR season in B0 was 159.3 kg·ha^−1^, which was significantly higher than the values obtained from other treatments (Fig. 5b). Compared to N_2_O, the cumulative CH_4_ uptake during the UT season increased with increasing rates of biochar amendment (Fig. 3c). The cumulative CH_4_ emission during the SR season decreased with an increasing rate of biochar application (Fig. 3d).

### Soil 1*6Sr DNA* and *pmoA* abundance

**Figure 6:**
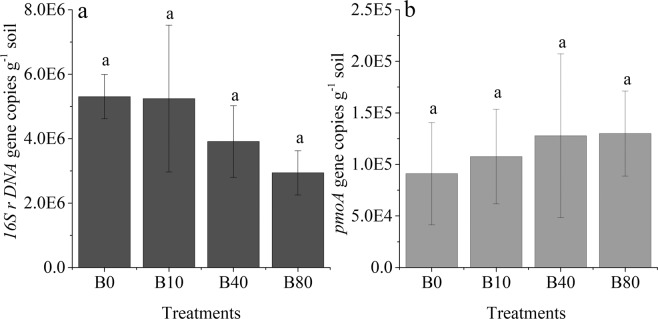
Methanogenic archaea *16Sr DNA* gene (**a**) and methanotroph *pmoA* gene (**b**) copy numbers in the rice growth period. Values with the same letter are not significantly different (p < 0.05); B0, B10, B40 and B80 are no biochar applied and biochar applied at the rate of 10, 40 and 80 t·ha^−1^, respectively.

**Figure 7:**
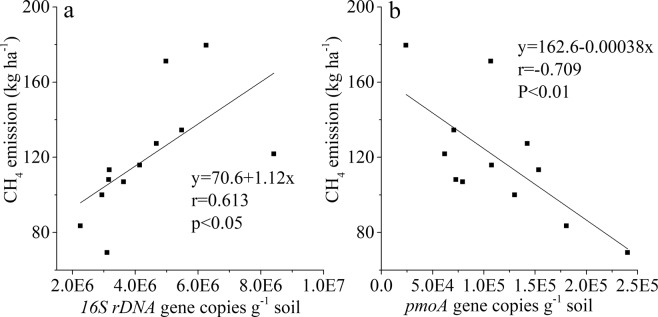
Relationship between the amount of CH_4_ emission during the rice growth period and methanogenic archaea *16Sr DNA* gene (**a**) and methanotroph *pmoA* gene (**b**) copy numbers.

Methanogenic archaea *16Sr DNA* and methanotroph *pmoA* gene copy numbers were determined after the rice harvest. Figure 6 shows that the methanogenic archaea *16Sr DNA* gene copy number in the B0 treatment was 5.3 × 10^6^ copies·g^−1^ soil; this decreased with biochar application, but there were no significant differences at *p* ≤ *0.05* due to large variations in the same treatments. The highest methanotroph *pmoA* gene copy numbers were found in the B80 treatment, and the lowest was found in the B0 treatment, with no significant difference recorded among the different treatments (Fig. 6). The cumulative CH_4_ emission during the SR season significantly increased with the methanogenic archaea *16Sr DNA* gene copy number and decreased significantly with the methanotroph *pmoA* gene copy number (Fig. 7).

## Discussion

### N_2_O emissions decreased with the rate of biochar application in both the UT and SR seasons

In contrast to our hypothesis that amendment with biochar may not reduce the N_2_O emission rate in acidic soil. We observed that N_2_O emissions decreased with the biochar application rate in both the UT and SR seasons (Fig. 3a,b). In line with the finding of Cayuela *et al*.^26^, there was a direct negative correlation between the rate of biochar application and N_2_O emission reductions. Both nitrification and denitrification have been identified as the predominant pathways for N_2_O production. It has been reported that biochar mitigation of N_2_O emissions via nitrification may be due to improvements in soil biological properties, physical properties, and chemical properties^11,20^. Furthermore, reducing nitrification substrate by NH_4_^+^ adsorption and the inhibition of potential microbial metabolic pathways (e.g. various polyphenolic and monoterpene compounds) play important roles in inhibiting nitrification and subsequent N_2_O emission^27,28^. Zhang *et al*.^29^ and Fidel *et al*.^30^ reported that amendment with biochar produced at 400 and 600 °C increased the NH_4_^+^ adsorption capacity by 62–81% and was maximized with low pyrolysis temperature (400 °C), leading to a significant decrease in soil inorganic N. The biochar used in our experiment was produced at 450 °C; thus, the adsorptive capacity for NH_4_^+^ related to nitrification could be a key factor in mitigating N_2_O emissions.

There are several mechanisms that have been suggested to explain the reasons for biochar mitigation of N_2_O emissions via denitrification. These mechanisms include NO_3_^−^ immobilization, aeration regulation and biochar toxicity^31^. It has been reported that adsorption and retention of NH_4_^+^ are improved in soils amended with biochar, indirectly leading to reductions in the amount of available N for denitrification, which is considered one of the important reasons for reducing N_2_O emissions via denitrification by biochar-amended soils^32^. Additionally, biochar has been proposed as a reducing agent for soils containing redox-reactive Fe(III) and Mn(IV) to compete with NO_3_^−^, reducing denitrification and promoting the reduction of N_2_O to N_2_^33^.

It has been reported that biochar can reduce the abundance of nitrite-reducing bacteria (carrying the *nirK* and *nirS* genes) but also increase the abundance of N_2_O-reducing bacteria (carrying the *nosZ* gene); thus, the mitigation of N_2_O emissions by biochar may be attributed to the fact that biochar can inhibit N_2_O production and simultaneously promote N_2_O consumption^14,34,35^. The reduction of N_2_O to N_2_ in the last step of denitrification may be improved, which would lead to a decrease in the N_2_O emission rate^13^. However, work performed by Ameloot *et al*.^36^ showed that a reduction in the N_2_O/(N_2_O + N_2_) ratio was not observed via the acetylene inhibition method, which suggested that biochar did not stimulate *nosZ* and the reduction of N_2_O to N_2_. These contradictory phenomena may be attributed to soil and biochar properties. It has been reported that biochar predominantly promotes the last step of denitrification in fine-textured soil^32^. In acidic soil, the abundance of nitrite-reducing bacteria and N_2_O-reducing bacteria increases as the soil pH is increased^37^. This may not only enhance the reduction of NO_3_^−^ to N_2_O but will also lead to stronger and more complete N_2_O reduction to N_2_, culminating in a balance in soil N_2_O emissions. Additionally, Cayula *et al*.^33,38^ suggested that the effect of biochar on the denitrification of N_2_O was mostly depend on its pH and the ratios of C/N and H/Corg.

In our observations, the soil pH was increased by biochar in the UT season from 5.4 to 6.1, whereas in the SR season, the pH returned to the initial range of values between 5.2 and 5.6 (Table 1). This was an indication that the addition of biochar to the soil had a rapid effect on soil pH, and this observation is in line with the findings of Castaldi *et al*.^39^. They reported that soil pH was significantly higher in soils treated with char incorporation than the control; however, the effect of biochar on pH was transient within the first three months, and the pH later returned to the initial value after 14 months^39^. As the soil pH was increased by biochar application, the cumulative N_2_O emissions from the treatments with different rates of biochar application were not significantly different because the inhibition of N_2_O emissions by biochar was likely offset by the stimulated pH increase.

### Biochar decreased CH_4_ emissions and increased CH_4_ uptake in the UT season and SR season

In contrast to our hypothesis that biochar would decrease CH_4_ emission in the first season and not in the second season, biochar decreased CH_4_ emissions in the second cropping (SR season). Similarly, Feng *et al*.^22^ and Chen *et al*.^40^ also observed a decline in CH_4_ flux following biochar application in paddy soils and suggested that the effects of biochar on CH_4_ emission were long-lived. However, contrary to this finding, Zhang *et al*.^41^ reported that CH_4_ emission was increased after biochar was added to paddies, while Xie *et al*.^42^ reported that there was no significant difference in seasonal cumulative CH_4_ emission between treatments. In the present study, we observed that the abundances of methanogenic archaea in the paddy were decreased by biochar application, while the methanotroph abundances were increased after biochar was applied, although the differences were not significant due to the large variations in a given treatment (Fig. 7). This showed that biochar addition decreased CH_4_ emissions, which may be due to decreased methanogenic archaea abundance; hence, CH_4_ could be utilized by methanotrophs. Feng *et al*.^22^ reported that methanogenic archaea were not inhibited by biochar amendments but there was a decreased ratio of methanogenic to methanotrophic microorganisms in paddy soils. An increase in methanotrophic abundance implies that methanogenic activity is been stimulated under biochar amendment (Fig. 7).

Biochar improved the sink capacity for CH_4_ (Figs. 3c and 5a), which may be directly attributed to the decrease in the bulk density and soil aeration that occurred during the UT season (Table 1). Environments with a CH_4_ sink capacity are suitable for methanotroph growth; however, Fungo *et al*.^11^ reported that biochar reduced the sink capacity of CH_4_. They attributed this to the easily mineralizable C provided by biochar, which was a substitute for methanotrophic bacteria^43^. Biochar acts as a slow C release source, and the relationship between biochar and C mineralization is dependent on the production procedure of the biochar^44^. The chemical properties and the type of biomass used for pyrolysis may also have influenced soil C and N cycling^45^. Additionally, biochar has high porosity, which may be able to increase soil aerobic micro-sites, affecting aeration and improving the supply and distribution of CH_4_ and O_2_^46,47^ When soil aeration increases as bulk density decreases (Table 1), the CH_4_ oxidation activity of methanotrophs is greatly enhanced, which results in CH_4_ emission mitigation^22,48^.

## Conclusion

Biochar application affected soil pH in the short term during the tobacco cropping season; however, in the saturated rice growth season, the pH reverted back to the initial value. The available potassium and SOM contents were improved, and BD was decreased by biochar application during the tobacco and rice growth seasons. N_2_O flux during the UT season was significantly greater than that in the saturated rice growth season and decreased with the rate of biochar application. The soils were sinks for CH_4_, and the cumulative CH_4_ uptake was increased with the rate of biochar application in the tobacco cropping season. However, there was considerable CH_4_ flux during the rice growth season, and the cumulative CH_4_ emission decreased with an increased rate of biochar application. Cumulative CH_4_ emissions had a negative relationship with methanotroph *pmoA* gene copy numbers and a positive relationship with methanogenic archaea *16Sr DNA* gene copy numbers in the soils, indicating that stimulating methanotrophs and depressing methanogenic archaea are the mechanisms for CH_4_ reduction upon biochar amendment. Therefore, to prevent environmental pollution and maintain the soil organic matter content, tobacco straw could be used as a biochar feedstock to reduce N_2_O and CH_4_ flux from soil. The rate of biochar application played important roles in N_2_O and CH_4_ flux, and further research should be conducted to study the relationship between biochar and the sink capacity for CH_4_.

## Materials and Methods

### Biochar production

Biochar was produced from dried tobacco straw; the straw was cut into small segments (<50 mm length) before being placed into the reactor. The reactor was heated by a step-wise procedure. The heating temperature was increased to 450 °C under anaerobic conditions and maintained for approximately 8 h. Before applying biochar to the field, the biochar was further reduced to smaller sizes of <5 mm. The concentrations of N, P, K, and organic C and pH (H_2_O) in the biochar were 15.0 g·kg^−1^, 1.4 g·kg^−1^, 20.1 g·kg^−1^, 475.92 g·kg^−1^ and 9.7, respectively.

### Field site description

The field experiment was conducted in 2015 in Jinan County, Fujian Province, China (119°36′86″E, 26°17′33″N). The mean annual temperature and precipitation in this region are 18.3 °C and 1500 mm (over 30 years), respectively, and the region is characterized by a subtropical monsoon climate. The soil is defined as an Anthrosol (WRB Soil Taxonomy), and the average concentrations of SOM, total N (TN), total phosphorus (TP), total potassium (TK), alkali-hydrolysable nitrogen (Av. N), available phosphorus (Av. P), and Av. K and pH (H_2_O) were 25.6 g·kg^−1^, 1.4 g·kg^−1^, 0.7 g·kg^−1^, 20.0 g·kg^−1^, 181.4 mg·kg^−1^, 58.0 mg·kg^−1^, 443.0 mg·kg^−1^ and 5.3, respectively. The cropping sequence at the site was as follows: tobacco was planted in mid-March, then rice was planted in mid-July, for more than 15 years. The root and straw of tobacco were removed before tilling by machine. The treatments included three rates of biochar application and a control that did not receive any biochar amendment: no biochar applied (B0); biochar applied at the rate of 10 t·ha^−1^ (B10); biochar applied at the rate of 40 t·ha^−1^ (B40); and biochar applied at the rate of 80 t·ha^−1^ (B80). Three replicate plots (24 × 6 m) of each treatment were established in a randomized block design. The biochar was applied on 1^st^ March 2015, before tobacco seedlings were transplanted. Except for the biochar, all treatments received N, P and K fertilizers at a recommended rate divided into three separate applications, which are given in Table 2. Compound fertilizers, urea and potassium nitrate were applied as N sources for tobacco; ammonium bicarbonate and urea were applied as N sources for rice.

**Table 2:** Annual fertilizer application rates in the field experiment (kg·ha^−1^).

Fertil- izers	UT season					SR season					Total amount
	Base fertilizers	Seedling stage	Rosette stage	Vigorous growth stage	Total	Base fertilizers	Green stage	Tillering stage	Heading stage	Total	
N	104.4	3.9	7.6	11.5	127.5	66.7	66.2	16.6	16.6	166.0	293.5
P_2_O_5_	97.0	—	1.0	1.0	99.0	29.9	—	—	—	29.9	128.9
K_2_O	266.4	13.3	23.3	74.2	377.2	36.0	36.0	—	—	72.0	449.2

UT is unsaturated tobacco cropping, and SR is saturated rice cropping.

### CH_4_ and N_2_O emission monitoring

Greenhouse gas emissions were monitored using static closed chambers^21^. Gas samples were collected between 9 and 11 am in a 7-day interval during the UT and SR seasons. Two chambers (0.5 m × 0.5 m × (0.5 + x) m) were placed on a fixed steel frame (stainless) in each plot after transplanting in the tobacco and rice growing seasons. One tobacco or six rice plants were closed in the chamber, and the height of the chamber was increased according to the height of the plant (x = 0.5 m or 1.0 m). To seal the rim of the chamber with a level surface, a groove (50 mm depth) along the top edge of each steel frame was filled with water. To minimize air temperature variation inside the chamber during the sampling period, the chambers were wrapped with a layer of porous insulation and aluminium foil. A circulating fan, humidity meter and temperature meter were equipped inside each chamber. After chamber closure, a syringe was used to collect gas samples at 0, 20, 40, and 60 min throughout the UT season and at 0, 10, 20, and 30 min during the SR season. The concentrations of N_2_O and CH_4_ were simultaneously analysed by a gas chromatograph (Agilent 7890B, USA), which was equipped with an electron capture detector (ECD) for N_2_O and a flame ionization detector (FID) for CH_4_ analysis^21^.

### Soil samples

Soil samples were collected before the experiment and after the tobacco and rice had been harvested for property analyses. Soil organic matter was analysed by wet digestion with H_2_SO_4_-K_2_Cr_2_O_7_, and total N was analysed using semi-micro Kjeldahl digestion with Se, CuSO_4_ and K_2_SO_4_ as catalysts^49^. A pH detector (Quark Ltd, Nanjing, China) was used to measure soil pH with a ratio of soil to water of 1:2.5 (*w/v*). Soil BD was analysed via the cutting ring method. Soil TP and TK were determined by the colorimetric and flame photometer methods after wet digestion with a mixture of H_2_SO_4_ and HClO_4_ and a mixture of HF and HClO_4_, respectively^50^. Soil Av. N was diffused with 1.0 mol·L^−1^ NaOH and trapped with 0.32 mol·L^−1^ H_3_BO_3_. Soil Av. P was extracted with a mixture of 0.025 mol·L^−1^ HCl and 0.03 mol·L^−1^ NH_4_F, while soil Av. K was determined by the 1.0 mol L^−1^ CH_3_COONH_4_ extraction method^51,52^.

A PowerSoil® DNA Isolation kit (MO BIO Laboratories, Inc., Carlsbad, USA) was used to extract DNA from 0.25 g fresh soil following the manufacturer’s instructions. The quality and quantity of DNA were checked by a NanoDrop spectrophotometer (NanoDrop Technologies Inc., Wilmington, USA). The copy number of methanogenic archaea *16Sr DNA* genes and methanotroph *pmoA* genes were enumerated by quantitative PCR using primer sets 1106 F/1378R^53^ and A189/m661^22^ with a CFX96 Optical Real-Time Detection System (Bio-Rad Laboratories, Inc. Hercules, CA). The qPCR standard was produced by plasmid DNA from representative clones including the methanogenic archaea *16Sr DNA* gene and methanotroph *pmoA* gene. The 25.0 µL reaction mixture contained 12.5 µL of SYBR Premix Ex Taq (TaKaRa Biotech, Dalian), 1.0 µL of each primer, 0.5 μL Rox Reference Dye II (50×), and 1.0 µL template. The thermal conditions of quantitative PCR for the methanogenic archaea *16Sr DNA* genes and methanotroph *pmoA* genes were those given by Feng *et al*.^22^ and Watanabe *et al*.^53^. Specific amplification of the *16Sr DNA* and *pmoA* genes was checked by confirming a single peak in melting-curve analysis. Copy numbers of genes are reported per dry weight of soil.

### Calculation

The rates of GHG emission from soil were calculated using Eq. (1), as follows:

1 $$F=\rho \times h\times dc/dt\times 273/(273+T)\times t$$

where *F* is the CH_4_ or N_2_O emission rate from soil (mg·m^−2^·h^−1^), *ρ* is the density of CH_4_ or N_2_O under standard atmospheric pressure (0.714 and 1.96 kg·m^−3^, respectively), *h* is the height of the static closed chambers (m), *dc/dt* is the rate of change in CH_4_ or N_2_O concentration, *T* is the temperature inside the chamber (°C), and *t* is the time of chamber closure (h).

The amounts of CH_4_ and N_2_O emissions were calculated using Eq. (2), as follows:

2 $$C=\mathop{\sum }\limits_{i=1}^{n}(\frac{{{F}}_{{i}}+{{F}}_{{i}+{1}}}{{2}}\times {24}\times D)$$

where *C* is the amount of CH_4_ or N_2_O emission (kg·ha^−1^), *F*_*i*_ *and F*_i + 1_ are the CH_4_ or N_2_O emission rates at times *i* and *i* + *1*, respectively (mg·m^−2^·h^−1^), and *D* is the number of days between times *i* and *i* + *1*.

### Statistical analysis

The differences in the rates and amounts of CH_4_ and N_2_O emissions and soil properties were examined by one-way ANOVA. The significant differences among treatments were identified by Duncan’s test (where *p* < *0.05*).
